# Blood glucose control and quality of health care in non-insulin-treated patients with Type 2 diabetes in Spain: a retrospective and cross-sectional observational study

**DOI:** 10.1111/j.1464-5491.2011.03258.x

**Published:** 2011-06

**Authors:** A Rodríguez, A Calle, L Vázquez, F Chacón, P Polavieja, J Reviriego, on behalf of the CADiNI Study Group

**Affiliations:** Clinical Research DepartmentLilly SA, Alcobendas; * Servicio de Endocrinología y Nutrición, Hospital Universitario San CarlosMadrid; † Servicio de Endocrinología y Nutrición, Hospital Universitario Marqués de ValdecillaSantander, Spain

**Keywords:** glycaemic control, glycated haemoglobin, hyperglycaemia, hypoglycaemic agents, insulin resistance

## Abstract

**Aims:**

To assess blood glucose control and quality of health care provided to non-insulin-treated patients with Type 2 diabetes mellitus in routine clinical practice in Spain.

**Methods:**

In this observational, retrospective, cross-sectional study, patients were grouped as either having good or suboptimal blood glucose control according to International Diabetes Federation or American Diabetes Association HbA_1c_ goals. Clinical and socio-demographic data and compliance with the main standard level of care recommendations of the International Diabetes Federation were recorded during a routine visit. Correlates of glucose control were analysed by logistic regression.

**Results:**

Many patients were grouped as having suboptimal control under International Diabetes Federation (61.9%) or American Diabetes Association (45.0%) criteria. The mean number of accomplished International Diabetes Federation recommendations (7.3 out of 11) was higher for endocrinologists (than for internists or primary care physicians), and significantly more patients under their care were in the good glucose control group (than with primary care physicians). More recommendations were associated with blood glucose control using International Diabetes Federation than American Diabetes Association criteria, demanding higher quality of health care for achieving stricter goals. Some recommendations were poorly observed, particularly those concerning patients’ education on diabetes, the prompt prescription of effective treatments and monitoring of complications. Diabetes complications were associated with being in the suboptimal control group. Patients’ education on diabetes and HbA_1c_ monitoring were associated with being in the good control group.

**Conclusions:**

These results demonstrate the need for improvement in the management of patients with non-insulin-treated Type 2 diabetes in actual clinical practice in Spain. Such improvement would entail a stricter adherence to International Diabetes Federation recommendations.

## Introduction

Type 2 diabetes mellitus is a chronic, progressive and heterogeneous disease. Recently, there has been a relevant increase of incidence of Type 2 diabetes and a noticeable proportion of patients are not achieving the currently established glycaemic goals [1]. Intensive research has shed light on further understanding of the disease, but the complexity of the underlying metabolic disorders largely impedes its adequate management. International and national diabetes organizations have developed evidence-based guidelines for optimal management of Type 2 diabetes [2–4]. Although both improving diabetes care and providing intensive blood glucose control therapies to patients with Type 2 diabetes increase management costs, they are cost-effective over a patient's lifetime because of the substantially reduced cost of complications, increased time free of complications and improved quality-adjusted life years [5,6]. It is thus crucial to search for effective strategies to improve the currently unsatisfactory clinical frame of Type 2 diabetes.

Controlling hyperglycaemia is one of the therapeutic goals [1,3,4], but it is well recognized that optimal management of Type 2 diabetes also requires overall metabolic control related to its multiple co-morbidities in order to prevent long-term macro- and microvascular complications [7–11].

Recommendations of care are thus directed toward adequate management of Type 2 diabetes disorders, as well as the prevention or amelioration of associated complications [2–4]. These recommendations include: education on diabetes for patients (providing knowledge of the disease and counselling for self-management); promotion for the achievement and maintenance of HbA_1c_ goals; appropriate application of lifestyle interventions and pharmacological therapies; monitoring of blood glucose and pressure levels, lipid profile and other cardiovascular risk factors; and routinely performing eye, kidney, feet and sensorimotor neuropathy examinations. All these recommendations are reasonably achievable in routine clinical practice and should be readily available to patients with Type 2 diabetes attending healthcare facilities.

This observational study investigated whether blood glucose control is related to the quality of health care provided to patients with non-insulin-treated Type 2 diabetes and also assessed other factors that might influence the achievement of current HbA_1c_ goals.

## Patients and methods

This nationwide, multi-centre, naturalistic, observational, retrospective, cross-sectional study evaluated patients with Type 2 diabetes in routine clinical settings in Spain. The investigators were endocrinologists, internists and primary care physicians with prior experience in clinical research who included patients in the same chronological order as they attended their outpatient clinics. To obtain a representative sample, the geographical distribution of study sites was proportional to the 2005 Spanish census and balanced among the three medical specialties concerned. During the 3-month inclusion period, retrospective (medical histories since diagnosis of Type 2 diabetes) and cross-sectional (the routine visit during which the patient was included in the study), socio-demographic and clinical data were recorded using structured case report forms.

This study complied with the International Conference on Harmonization's Good Clinical Practice guidelines and Spanish regulatory requirements. The protocol was approved by an accredited institutional ethics committee. All patients signed and dated written informed consent forms.

The inclusion criteria for patients were: male or female; aged ≥ 30 years; diagnosed with Type 2 diabetes according to the American Diabetes Association (ADA) guidelines [12]; not being treated with insulin; and having clinical records available at the study centres. Pregnant women and patients with a diabetes type other than Type 2 [12] were not eligible.

The primary objective was to evaluate the blood glucose control of patients with non-insulin-treated Type 2 diabetes in relation to the quality of health care they received, determined by the accomplishment of the main International Diabetes Federation (IDF) recommendations for the standard level of care [2]. Patients were grouped as achieving good blood glucose control [≤ 6.5% (≤ 48 mmol/mol) [13] or ≤ 7.0% (≤ 53 mmol/mol) [13]] vs. suboptimal blood glucose control [> 6.5% (> 48 mmol/mol) or > 7.0% (> 53 mmol/mol)] according to IDF [2] or ADA [4] HbA_1c_ goals, respectively. Secondary objectives were to evaluate different features of these groups: (i) socio-demographic and clinical data; (ii) therapeutic regimens; (iii) metabolic control; (iv) prevalence of diabetes complications; and (v) distribution by the type of specialist providing health care.

**FIGURE 1:**
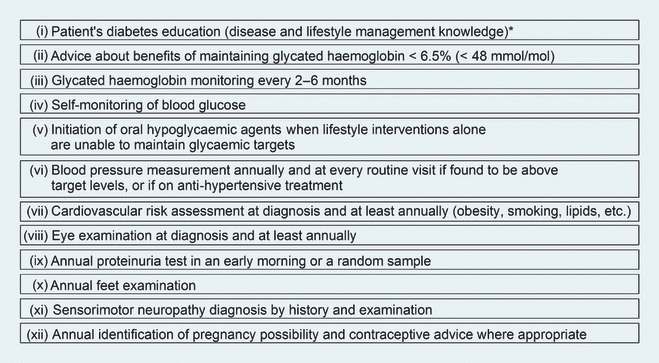
International Diabetes Federation's recommendations for standard care assessed in this study. *Diabetes education refers to participation in non-evaluated, structured training sessions about diabetes course and complications, the therapeutic options available and self-care strategies that improve general therapeutic effectiveness. These sessions were conducted by a specialized nurse and, less frequently, by a specialised physician. Because there was not a protocol for these sessions, their contents varied from one site to another.

The IDF recommendations for standard level of care assessed in this study are listed in Fig. 1. The IDF's recommendation regarding the initiation of insulin therapy was not included because insulin-treated patients were not eligible. However, the number of patients who initiated insulin therapy at the cross-sectional visit was recorded.

### Statistical analyses

At least 2170 patients were required (1085 patients each group), assuming that patients were evenly distributed (50%) between the group with good blood glucose control and the group with suboptimal blood glucose control. This sample size would detect ≥ 6% between-group differences in the proportions accomplishing the IDF standard level of care recommendations, with 80% statistical power and a 5% significance level. The participation of 250 investigators, recruiting 10 patients each, was planned to compensate for missing or non-evaluable data.

Data were summarized using descriptive statistics. The recommendations for cardiovascular risk assessment and eye examinations were considered accomplished if performed at least annually. The time to the first change of pharmacological therapy was estimated by the Kaplan–Meier method.

Two logistic regression analyses were performed to evaluate which factors might relate to being in either group, using good vs. suboptimal blood glucose control as the dependent variable, under either IDF or ADA criteria. The independent variables were the compliance with the 11 main IDF standard level of care recommendations (yes vs. no) and some derived from the socio-demographic, clinical and healthcare data, including physicians’ specialties, that, in prior bivariate analyses regarding glycaemic control, showed a < 10% significance level. Different models were tested progressively to exclude those independent variables with the highest *P*-values and which were confirmed as non-significant by the likelihood ratio test.

Missing data from the variables required to evaluate the primary objective were imputed by the worst-case method as follows: (i) the last value of HbA_1c_ was used and patients with no available data within the previous 6 months were placed in the suboptimal blood glucose control group and (ii) missing data on an IDF recommendation were imputed as not accomplished. Inferences were made at a 5% significance level. SAS version 8.02 software (SAS Institute, Cary, NC, USA) was used for the statistical analyses.

Sensitivity analyses evaluated possible differences between the population of patients with no missing data for the variables required to assess the primary objective (*n* = 1514, observed population) and the population of patients included per protocol (*n* = 2266, total population).

## Results

**FIGURE 2:**
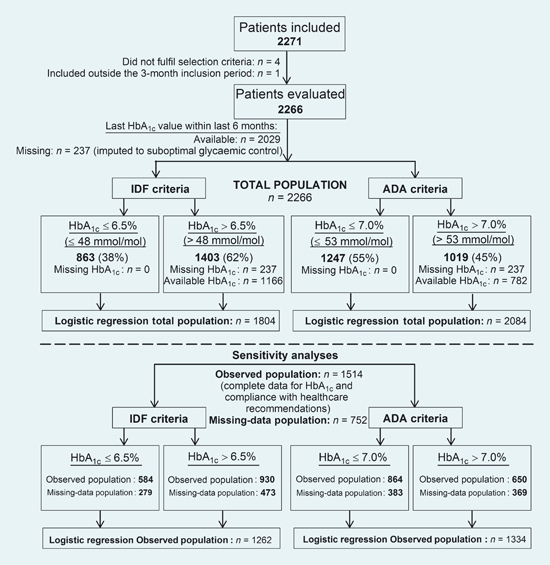
Patient disposition. ADA, American Diabetes Association; IDF, International Diabetes Federation.

Between March and May 2007, 220 investigators collected data from 2271 patients. Investigators were endocrinologists (37%), internists (31%) and primary care physicians (32%). Data from 2266 patients were analysed. Patients’ disposition is depicted in Fig. 2 and their characteristics are summarized in Table 1 (by blood glucose control) and Table 2 (by medical specialty). A number of patients were grouped as having suboptimal blood glucose control by IDF (61.9%) or ADA (45.0%) criteria. Proportions of patients in the suboptimal blood glucose control group were higher among those treated by primary care physicians (64.5/50.6%, IDF/ADA criteria), followed by those treated by internists (60.6/43.5%) and endocrinologists (60.8/41.4%). Nearly all patients (93.9%) were treated with both diet and pharmacological therapy. The oral hypoglycaemic agents most frequently prescribed were metformin and glibenclamide for the first received treatment and metformin and glimepiride for the second treatment, both alone (monotherapy) or in combination. Less than 10% patients received other treatments. The time to the first change of pharmacological therapy was similar, but slightly longer, in patients in the good vs. suboptimal blood glucose control group (respective median Kaplan–Meier point estimates: 3.9 vs. 3.5 years for IDF criteria and 3.7 vs. 3.4 years for ADA criteria).

**Table 1:** Patient and Type 2 diabetes treatment characteristics according to blood glucose control per the International Diabetes Federation (IDF) and the American Diabetes Association (ADA) criteria

	HbA_1c_ IDF criteria		HbA_1c_ ADA criteria	
		
	≤ 6.5%(≤ 48 mmol/mol) (*n* = 863)	> 6.5%(> 48 mmol/mol) (*n* = 1403)	≤ 7.0%(≤ 53 mmol/mol) (*n* = 1247)	> 7.0%(> 53 mmol/mol) (*n* = 1019)
Age, years, mean (sd)	64.1 (11.0)	64.6 (11.2)	64.2 (10.9)	64.6 (11.5)
Years since diagnosis, mean (sd)	6.8 (6.6)	7.9 (6.4)	7.1 (6.5)	7.9 (6.5)
Female gender, *n*/*N*‡ (%)	400/863 (46.3)	671/1403 (47.8)	584/1247 (46.8)	487/1019 (47.8)
Body mass index, kg/m^2^, mean (sd)	29.9 (5.3)	30.2 (5.2)	29.9 (5.3)	30.3 (5.1)
Type 2 diabetes family history, *n*/*N*‡ (%)	461/767 (60.1)	789/1230 (64.1)	685/1109 (61.8)	565/888 (63.3%)
Race*				
Caucasian, *n*/*N*‡ (%)	811/862 (94.1)	1306/1399 (93.4)	1168/1245 (93.8)	949/1016 (93.4)
Latin American/others, *n*/*N*‡ (%)	51/862 (5.9)	93/1399 (6.6)	77/1245 (6.2)	67/1016 (6.6)
Academic level*				
Without any grade certificate, *n*/*N*‡ (%)	147/863 (17.0)	323/1400 (23.1)	217/1247 (17.4)	253/1016 (24.9)
Primary, *n*/*N*‡ (%)	389/863 (45.1)	668/1400 (47.7)	571/1247 (45.8)	486/1016 (47.8)
Secondary/higher, *n*/*N*‡ (%)	326/863 (37.8)	409/1400 (29.2)	458/1247 (36.7)	277/1016 (27.3)
Smoker/ex-smoker, *n*/*N*‡ (%)	307/860 (35.7)	507/1399 (36.2)	457/1243 (36.8)	357/1016 (35.1)
Hypertension, *n*/*N*‡ (%)	547/863 (63.4)	933/1403 (66.5)	801/1247 (64.2)	679/1019 (66.6)
SBP, mmHg, mean (sd)	134.6 (16.7)	137.6 (17.4)	135.1 (16.5)	138.1 (18.0)
DBP, mmHg, mean (sd)	76.8 (9.8)	78.4 (10.0)	77.2 (9.7)	78.5 (10.2)
Dyslipidaemia, *n*/*N*‡ (%)	541/863 (62.7)	923/1403 (65.8)	780/1247 (62.6)	684/1019 (67.1)
LDL cholesterol, mmol/l, mean (sd)	2.9 (0.8)	3.0 (0.9)	2.9 (0.8)	3.0 (0.9)
HDL cholesterol, mmol/l, mean (sd)	1.3 (0.5)	1.3 (0.6)	1.3 (0.5)	1.3 (0.6)
Triglycerides, mmol/l, mean (sd)	1.6 (1.1)	1.8 (1.1)	1.6 (1.1)	1.8 (1.2)
Diabetes complications, *n*/*N*‡ (%)	266/860 (30.9)	522/1395 (37.4)	403/1240 (32.5)	385/1015 (37.9)
First HbA_1c_ value, %, mean (sd)	7.1 (1.6)	7.8 (1.6)	7.2 (1.5)	7.9 (1.6)
mmol/mol, mean (sd)†	54 (17)	62 (17)	55 (16)	63 (17)
Last HbA_1c_ value, %, mean (sd)	5.9 (0.6)	7.6 (1.0)	6.2 (0.6)	8.0 (1.0)
mmol/mol, mean (sd)†	41 (7)	60 (11)	44 (7)	64 (11)
Last FPG value, mmol/l, mean (sd)	7.1 (1.6)	8.6 (2.4)	7.2 (1.6)	8.9 (2.5)
Current Type 2 diabetes treatment*				
No treatment, *n*/*N*‡ (%)	1/863 (0.1)	1/1403 (0.1)	1/1247 (0.1)	1/1019 (0.1)
Diet only, *n*/*N*‡ (%)	60/863 (7.0)	45/1403 (3.2)	73/1247 (5.9)	32/1019 (3.1)
Drugs only, *n*/*N*‡ (%)	5/863 (0.6)	27/1403 (1.9)	10/1247 (0.8)	22/1019 (2.2)
Diet + drugs, *n*/*N*‡ (%)	797/863 (92.4)	1330/1403 (94.8)	1163/1247 (93.3)	964/1019 (94.6)
At least one treatment change, *n*/*N*‡ (%)	345/702 (49.1)	733/1165 (62.9)	537/1018 (52.8)	541/849 (63.7)
Monotherapy as first treatment, *n*/*N*‡ (%)	611/702 (87.0)	1027/1165 (88.2)	900/1018 (88.4)	738/849 (86.9)
Monotherapy as second treatment, *n*/*N*‡ (%)	83/324 (25.6)	157/711 (22.1)	123/509 (24.2)	117/526 (22.2)
HbA_1c_ at start of diet, %, mean (sd)	7.2 (1.6)	7.9 (1.5)	7.4 (1.5)	8.0 (1.6)
mmol/mol, mean (sd)†	55 (17)	63 (16)	57 (16)	64 (17)
HbA_1c_ at start of OHA monotherapy, %	7.3 (1.4)	8.1 (1.4)	7.5 (1.3)	8.2 (1.4)
mmol/mol, mean (sd)†	56 (15)	65 (15)	58 (14)	66 (15)
HbA_1c_ at start of two OHAs combined, %	7.7 (1.4)	8.1 (1.2)	7.7 (1.2)	8.3 (1.2)
mmol/mol, mean (sd)†	61 (15)	65 (13)	61 (13)	67 (13)
HbA_1c_ at start of three OHAs combined, %	7.8 (1.2)	8.2 (1.0)	7.9 (1.1)	8.3 (1.0)
mmol/mol, mean (sd)†	62 (13)	66 (11)	63 (12)	67 (11)
Started insulin at study visit, *n*/*N*‡ (%)	12/852 (1.4)	139/1362 (10.2)	20/1227 (1.6)	131/987 (13.3)
Accomplished IDF recommendations, mean (sd)	7.3 (2.3)	7.2 (2.3)	7.4 (2.3)	7.1 (2.3)

* Stratified variables; percentages add up to 100% within each group.

† Transformed values for HbA_1c_. The original values (in %) are in the row immediately above.

‡ N Number of patients with data available.

DBP, diastolic blood pressure; FPG, fasting plasma glucose; OHA, oral hypoglycaemic agent; SBP, systolic blood pressure.

**Table 2:** Patient and characteristics of Type 2 diabetes treatment according to the medical specialty of the treating physician

	Endocrinology (*n* = 841)	Internal medicine (*n* = 703)	Primary care (*n* = 722)
Age, years, mean (sd)	62.4 (10.8)	66.3 (11.0)	65.0 (11.4)
Years since diagnosis, mean (sd)	8.5 (7.2)	7.2 (6.4)	6.6 (5.5)
Female gender, *n*/*N*‡ (%)	417/841 (49.6)	311/703 (44.2)	343/722 (47.5)
Race*			
Caucasian, *n*/*N*‡ (%)	813/836 (97.3)	659/703 (93.7)	645/722 (89.3)
Latin American/others, *n*/*N*‡ (%)	23/836 (2.8)	44/703 (6.2)	77/722 (10.7)
Academic level*			
Without any grade certificate, *n*/*N*‡ (%)	144/840 (17.1)	136/703 (19.4)	190/720 (26.4)
Primary, *n*/*N*‡ (%)	383/840 (45.6)	335/703 (47.7)	339/720 (47.1)
Secondary/higher, *n*/*N*‡ (%)	312/840 (37.1)	232/703 (33.0)	191/720 (26.5)
Smoker/ex-smoker, *n*/*N*‡ (%)	298/836 (35.7)	273/702 (38.9)	243/721 (33.7)
Hypertension, *n*/*N*‡ (%)	514/841 (61.1)	498/703 (70.8)	468/722 (64.8)
SBP, mmHg, mean (sd)	135.6 (18.0)	138.1 (18.5)	135.9 (14.8)
DBP, mmHg, mean (sd)	77.3 (9.7)	78.6 (10.9)	77.5 (9.1)
Dyslipidaemia, *n*/*N*‡ (%)	552/841 (65.6)	471/703 (66.7)	441/722 (61.1)
LDL cholesterol, mmol/l, mean (sd)	2.8 (0.8)	2.9 (0.9)	3.1 (0.8)
HDL cholesterol, mmol/l, mean (sd)	1.3 (0.4)	1.2 (0.4)	1.3 (0.7)
Triglycerides, mmol/l, mean (sd)	1.6 (1.0)	1.8 (1.3)	1.7 (1.1)
Diabetes complications, *n*/*N*‡ (%)	304/837 (36.2)	284/700 (40.6)	200/718 (27.9)
Last HbA_1c_ value, %, mean (sd)	6.9 (1.1)	6.9 (1.3)	6.8 (1.1)
mmol/mol, mean (sd)†	52 (12)	52 (14)	51 (12)
Last FPG value, mmol/l, mean (sd)	8.0 (2.2)	8.0 (2.4)	7.9 (2.0)
Current Type 2 diabetes treatment*			
No treatment, *n*/*N*‡ (%)	1/841 (0.1)	0/703 (0.0)	1/722 (0.1)
Diet only, *n*/*N*‡ (%)	20/841 (2.4)	28/703 (4.0)	57/722 (7.9)
Drugs only, *n*/*N*‡ (%)	13/841 (1.6)	14/703 (2.0)	5/722 (0.7)
Diet + drugs, *n*/*N*‡ (%)	807/841 (96.0)	661/703 (94.0)	659/722 (91.3)
At least one treatment change, *n*/*N*‡ (%)	450/697 (64.6)	334/594 (56.2)	294/576 (51.0)
Monotherapy as first treatment, *n*/*N*‡ (%)	573/697 (82.2)	538/594 (90.6)	527/576 (91.5)
Accomplished IDF recommendations, mean (sd)	8.0 (2.1)	6.8 (2.5)	6.9 (2.2)

* Stratified variables; percentages add up to 100% within each group.

† >Transformed values for HbA_1c_. The original values (in %) are in the row immediately above.

‡ N Number of patients with data available.

DBP, diastolic blood pressure; FPG, fasting plasma glucose; IDF, International Diabetes Federation; SBP, systolic blood pressure.

More patients with secondary or higher academic levels were in the good vs. suboptimal control group, whereas the opposite occurred among patients without any academic grade (Table 1). Interestingly, historical HbA_1c_ levels were higher among patients in the suboptimal blood glucose control group at each treatment change (Table 1). When the ADA criteria for blood glucose control were applied to define the groups instead, the results were quite similar to those provided for the groups formed by IDF criteria, with the exception that the proportion of patients in the ADA good control group with diabetes complications was greater, as were the mean HbA_1c_ values in both groups (first, last and those at each treatment change), corresponding to the higher cut-off value using the ADA criteria. Irrespective of glycaemic control, mean values of HDL cholesterol and triglycerides were within the normal ranges, but LDL cholesterol was above optimal level. More than two-thirds of patients were hypertensive.

Patients’ characteristics differed among medical specialties also (Table 2). Patients treated by primary care physicians had shorter diabetes duration, lower academic level, were more frequently from a non-Caucasian origin and treated with diet alone and had fewer diabetes complications than those treated by other specialists. Endocrinologists changed diabetes treatments more frequently.

**FIGURE 3:**
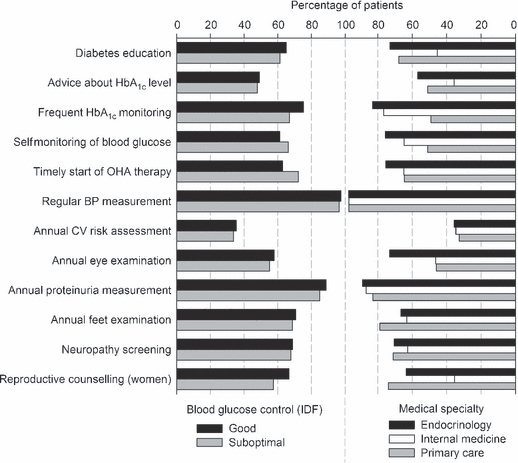
Compliance with IDF recommendations according to glycaemic control per the IDF criteria and to the treating physician's specialty. BP, blood pressure; CV, cardiovascular; IDF, International Diabetes Federation; OHA, oral hypoglycaemic agent.

Compliance with IDF standard level of care recommendations is presented in Fig. 3. Compliance was higher for recommended blood pressure measurement (96.8%), with similar fulfilment in the two groups, and annual proteinuria test (86.3%), which was accomplished by more patients in the good blood glucose control group. Cardiovascular risk-factor assessment (34.3%) and advice about benefits of HbA_1c_ level ≤ 6.5% (≤ 48 mmol/mol) (48.3%) had the lowest compliance rates. The other recommendations showed compliance rates ranging between 56.2 and 70.2%. With the exception of self-monitoring of blood glucose and the start of oral hypoglycaemic agent therapy when diet alone is insufficient, all recommendations were fulfilled by a greater proportion of patients in the good control group than in the suboptimal control group. The mean number of accomplished recommendations was 7.3 out of 11; by specialties, patients treated by endocrinologists fulfilled more (Table 2, Fig. 3). Delivery of structured education programmes on diabetes, advice on HbA_1c_ and reproductive counselling were comparatively scarce among patients treated by internists, and a low proportion of patients treated by primary care physicians had documentation of regular and frequent HbA_1c_ monitoring or self-monitoring of blood glucose.

Sensitivity analyses showed similar socio-demographic and clinical data in the total and observed populations (data not shown). The differences were minimal regarding blood glucose control grouping, indicating that slightly more patients qualifying for the good blood glucose control group were in the observed population (57.1%) than in the total population (55.0%), which was expected after imputing the data using the worst-case method.

Results of logistic regression analyses are illustrated in Table 3. Because HbA_1c_ values at or near diabetes diagnosis were not available for many patients, this variable was excluded from the models. These analyses revealed that patients receiving education on diabetes, undergoing regular HbA_1c_ determinations, having secondary or higher academic levels and being treated with diet alone had a greater likelihood of being in the good blood glucose control group under IDF criteria. Only HbA_1c_ monitoring indicated a greater likelihood of being in the good control group under ADA criteria. Conversely, patients starting oral hypoglycaemic agent therapy, receiving advice about the importance of maintaining HbA_1c_≤ 6.5% (≤ 48 mmol/mol) and performing self-monitoring of blood glucose had a lower likelihood of being in the good glycaemic control group. Also demonstrating a lower likelihood of good glycaemic control (by IDF and/or ADA criteria) were patients with: a family history of Type 2 diabetes; higher systolic blood pressure, fasting plasma glucose and total cholesterol levels; diabetes complications; or treatment by primary care physicians. In the observed population (data not shown), associations were less evident. There were fewer patients in the suboptimal blood glucose control group in this population because no data were imputed by the worst-case method.

**Table 3:** Results of the logistic regression of having good blood glucose control as per the criteria of the International Diabetes Federation (IDF) and the American Diabetes Association (ADA)

Independent variables†	IDF (HbA_1c_≤ 6.5 vs. > 6.5%) (≤ 48 vs. > 48 mmol/mol) OR (95% CI)§	ADA (HbA_1c_≤ 7.0 vs. > 7.0%) (≤ 53 vs. > 53 mmol/mol)OR (95% CI)§
Standard care (yes vs. no)‡		
Education on diabetes	1.54 (1.10–2.14)*	1.15 (0.84–1.56)
Advice about HbA_1c_ level	0.65 (0.47–0.90)*	1.02 (0.76–1.38)
Frequent HbA_1c_ monitoring (2–6 months)	1.39 (1.05–1.84)*	1.59 (1.26–2.00)*
Self monitoring blood glucose	0.73 (0.58–0.92)*	0.85 (0.69–1.06)
Timely start of OHA therapy	0.50 (0.39–0.63)*	0.62 (0.50–0.77)*
Regular blood pressure measurement	1.19 (0.59–2.40)	1.02 (0.58–1.80)
Annual CV risk assessment	1.03 (0.82–1.29)	0.95 (0.77–1.17)
Annual eye examination	1.00 (0.79–1.27)	0.91 (0.73–1.13)
Annual proteinuria measurement	1.30 (0.90–1.86)	1.38 (0.99–1.90)
Annual feet examination	1.14 (0.83–1.57)	0.93 (0.69–1.24)
Neuropathy screening	1.02 (0.74–1.39)	1.13 (0.85–1.51)
Primary care physician vs. endocrinologist	0.77 (0.58–1.03)	0.69 (0.53–0.89)*
Internist vs. endocrinologist	0.99 (0.76–1.30)	0.98 (0.76–1.25)
Secondary/higher vs. other academic background	1.29 (1.03–1.61)*	1.23 (0.99–1.52)
Time from diabetes diagnosis	0.99 (0.97–1.00)	—
Type 2 diabetes family history (yes vs. no)	0.80 (0.64–1.00)*	—
Last FPG value	0.98 (0.97–0.98)*	0.98 (0.97–0.98)*
Last SBP value	0.99 (0.98–0.99)*	0.99 (0.99–1.00)
Last total cholesterol value	—	0.99 (0.99–0.99)*
Number of blood lipid measurements in the last 2 years	1.05 (1.00–1.11)	—
Diet alone vs. diet + drug therapy	1.70 (1.03–2.81)*	1.58 (0.96–2.58)
Drug alone vs. diet + drug therapy	0.40 (0.13–1.22)	0.35 (0.14–0.85)*
Diabetes complications (present vs. absent)	0.82 (0.65–1.04)	0.78 (0.63–0.96)*
CV risk factors (present vs. absent)	0.70 (0.41–1.22)	—

* Significant association.

† For categorical variables, the last category is the reference category.

‡ Accomplishment of one of the IDF recommendations was not included in the logistic regression because it only concerned women.

§ An odds ratio > 1 indicates a greater chance of having good glycaemic control; an odds ratio < 1 indicates a lower chance of having good glycaemic control.

CV, cardiovascular; FPG, fasting plasma glucose; OHA, oral hypoglycaemic agent; SBP, systolic blood pressure.

## Discussion

This observational study evaluated abundant socio-demographic and clinical data from patients with non-insulin-treated Type 2 diabetes in outpatient practices in Spain. Blood glucose control was suboptimal in approximately one in two patients, reinforcing the notion that adequate control is very difficult to achieve [4]. Compliance with the IDF recommendations for the standard level of care was moderate and only approximately one in ten patients with suboptimal control started insulin therapy at the cross-sectional evaluation. A higher proportion of patients with good blood glucose control accomplished a majority of the recommendations. Regression analyses confirmed the association between blood glucose control and accomplishment of care recommendations and also revealed associations between patients’ academic backgrounds and the settings where their care takes place (primary vs. specialized outpatient consultations).

Because two different cut-offs were analysed, this research can provide some comparative data on how the different HbA_1c_ goals relates to the quality of health care. The present results suggest that the stricter the glycaemic goal used for defining adequate control, the stronger the association between the quality of health care and blood glucose control. The most recent editions of therapeutic recommendations advocate the use of flexible goals to the control of hyperglycaemia [4], based in part on the dissipation of the concerns regarding the risk–benefit ratio of intensified blood glucose control. This study adds that the stricter adherence to therapeutic recommendations, including the use of insulin therapy, might serve to improve blood glucose control among patients with Type 2 diabetes.

The relevant role of HbA_1c_ monitoring has been consistently recognized in parallel with the long-term benefits of HbA_1c_ control [1,3,4,9,11]. This study contributes evidence regarding its actual value under routine clinical practice conditions in a non-selected and heterogeneous sample of patients with Type 2 diabetes. Less clear, however, is the relationship between education on diabetes and blood glucose control. Benefits that are significant only in the short term and are restricted to selected subgroups of patients have been reported [14,15], albeit factors such as the longer duration of programmes and a higher frequency of face-to-face patient-educator contacts have been noted to favour positive outcomes [15,16]. The results reported here support the generic benefit of education on diabetes, despite the heterogeneity of programmes used, underscoring its role in improving outcomes across the diverse range of patients included in this sample. It also supports the notion that psychological barriers, which are mainly related to the strictness of the diabetes regimen and are responsive to educational techniques, are relevant impediments to the implementation of diabetes care [17,18]. Compared with the difficulties in deploying and maintaining lifestyle changes in primary diabetes prevention trials [19], the present results suggest that the benefit of education on diabetes is more certain in patients with Type 2 diabetes than in individuals in the pre-diabetic range. As mentioned, the programmes used here were not homogeneous. It is thus feasible that even better results would be expected if a uniform protocol for diabetes education was developed and deployed in Spain, as occurred in other countries [20]. Lastly, together with education on diabetes, there was an association between higher academic background and good blood glucose control. Although academic level differs from education on diabetes, it is reasonable to think that more literate patients might learn more easily or be more knowledgeable about diabetes.

The paradoxical association between starting oral hypoglycaemic agent therapy when lifestyle interventions alone are insufficient, performing self-monitoring of blood glucose and advising patients regarding the benefits of achieving glycaemic targets with suboptimal blood glucose control does not necessarily indicate a negative contribution by these recommendations. Patients with suboptimal control showed consistently higher HbA_1c_ values from the point of disease diagnosis, with negligible variation among them (contrasting with relevant reductions in patients with good blood glucose control), and more of those patients needed oral hypoglycaemic agent therapy, including more therapeutic changes and initiation of insulin therapy. Higher HbA_1c_ values at each treatment change might suggest a faster disease progression or greater resistance to treatments, despite the efforts to augment treatment when control is inadequate. Following this reasoning, patients with suboptimal control would have required more insistent guidance regarding blood glucose control awareness than patients consistently meeting glycaemic goals, hence explaining the relationship between these factors and suboptimal blood glucose control. Yet this does not imply that compliance with IDF recommendations was optimal. If there is a subset of patients exhibiting distinct and more aggressive disease progression, for example a rapid loss of B-cell function, efforts should be made to promptly identify and intensify therapeutic measures, as they may be of particular benefit in these individuals [21].

The lower likelihood of good blood glucose control of patients treated by primary care physicians with respect to those treated by endocrinologists, despite having a shorter duration of diabetes, raises a concern, because the majority of Type 2 diabetes care is delivered at the primary care level. Structural factors that have been identified as affecting the quality of diabetes care by primary care physicians in the USA more than 10 years ago (such as lack of time and other resources to perform recommended procedures, or a busy primary clinic) [22] in all probability currently exist in Spain and in other European countries. In addition to the technical quality of care, which was the focus of this study, environmental factors and other external determinants, the so-called service quality, may also affect the global quality of health care [23]. Different approaches for improving the quality of Type 2 diabetes in primary care have shown positive outcomes [6,24,25]. The fact that patients treated by endocrinologists, compared with those treated by primary care physicians, had a longer disease progression and more diabetes complications despite being younger suggests that they feature a more challenging clinical profile. It is feasible that endocrinologists preferentially treat patients who are referred because they require a more thorough evaluation and care. Yet their better achievement of glycaemic goals highlights that there is considerable room for improvement in the primary care setting. The interventions made by endocrinologists to improve control in patients with a progressed disease might be of greater help if they were carried out at an earlier stage [7]. Given the potential long-term benefits of delaying or preventing diabetes complications by early interventions, further research on this topic is required.

Failure to comply with blood pressure and lipid targets was common, as denoted by the mean values of systolic blood pressure and LDL cholesterol above their normal ranges. Better management of hypertension and dyslipidaemia, together with blood glucose control, is recommended provided that substantial reductions in cardiovascular risk can be achieved [4,8]. The achievement of HDL cholesterol targets, in contrast with reports from other settings [26], might be a distinct feature of this sample related to Mediterranean dietary habits. Prior investigations on the Spanish sample of the Diabetes Nutrition and Complications Trial (DNCT) also showed optimal HDL cholesterol and triglyceride levels, which might be explained by the observance of healthy unsaturated to saturated fatty acid ratios [27,28], despite poor global adherence to nutritional recommendations [27–29]. This is an issue of interest because stronger associations have been suggested between fatty acid ratios in the diet and deferral of complications than with absolute unsaturated fatty acid consumption [27].

This study has strengths and limitations. Its large sample size and the inclusion of different specialists’ care provide a reliable representation of the Spanish patients with non-insulin-treated Type 2 diabetes. Conversely, the national scope of the study being restricted to Spain prevents international extrapolation of the results, particularly to countries with different dietary habits, which is a significant influence on diabetes outcomes [1,4]. The retrospective character of the study also constitutes a limitation, mainly attributable to the lack of certain management details (e.g. intervals between treatment changes), more precise information about dietary patterns of patients and the large amount of missing data for HbA_1c_ values at diabetes diagnosis.

In conclusion, this study has shown there is insufficient compliance with the IDF standard level of care recommendations for Type 2 diabetes in real clinical practice in Spain. With approximately half of patients failing to achieve adequate blood glucose control, a stricter adherence to these recommendations, particularly in the primary care setting, is recommended based on this investigation, provided that they are associated with improved metabolic control.

## Abbreviations

ADA: American Diabetes Association
IDF: International Diabetes Federation

## Supporting Information

**Appendix S1.** List of investigators who participated in the Quality of Health Care Assessment of Patients with Type 2 diabetes Non-Insulin-Treated study (the CADiNI Study) in alphabetical order by geographical location (province).
