# Supplementary material for: Blood glucose control and quality of health care in non-insulin-treated patients with Type 2 diabetes in Spain: a retrospective and cross-sectional observational study
Source: Diabet Med. 2011 Jun;28(6):731–40. doi: 10.1111/j.1464-5491.2011.03258.x (PMC3123709; doi:10.1111/j.1464-5491.2011.03258.x)
Supplement: Supplementary file 1 [file dme0028-0731-SD1.doc]

**Appendix**

Investigators that participated in the CADiNI (*Quality of Health* ***C****are* ***A****ssessment of patients with type 2* ***Di****abetes* ***N****on-****I****nsulin-treated*) Study are listed in alphabetical order by geographical location (province):

**A Coruña:** Bellido D., Ferrón F., Soto A., Suárez M.; **Albacete:** López L.M.; **Alicante:** Ferriz P., López P.J., Ruiz J.M., Vences L.F., **Almería:** Cepero D.; **Asturias:** Casal F., Díaz J.B., Lorente J.J., Varela M.C.; **Ávila:** Barragán J.M.; **Badajoz:** Carramiñana F.C., Rodríguez C., Sillero A.; **Barcelona:** Auladell M.A., Carrasco A., Casamayor R., Creixell J., Estepa A., Fandos J.M., Hernández R., Méndez A., Monfort F., Murillo J.I., Mussoll J., Nieto V., Noguera R., Ramentol A., Sáenz G., Sanclemente C., Sauque J., Vericat A.; **Burgos:** Cancelo P., Castillo L.A., Dueñas C., Huidobro J.A.; **Cáceres**: Antón J., Fernández J., Fraile J.; **Cádiz**: Álvarez J.A., Escribano J., Galán A., García J.R., Ingelmo R., López A.; **Cantabria**: Huidobro C., Pazos F., Vázquez L.A., Zarrabeitia R.; **Castellón**: Cano P., Garzón S., González C., Llopis J.J.; **Ciudad Real:** Chamorro R., Ocaña C., Reales P.; **Córdoba:** Calañas A., Gálvez A.F., Paniagua-González J.A.; **Cuenca:** Dimas J.F., Gómez C., Toldos G.; **Girona:** Esteve E., Grau A.J., Martín-Urda A.I.; **Granada:** Jerónimo R., Bueno M.A., León B., Pechuán J., Piédrola G., Quesada M., Sancho F., Tejeda P.; **Huelva:** Arazola J.A., Rivas G., Roldán E.; **Islas Baleares:** Arnáiz C., Gutiérrez-Garzarain A., Moll M.G., Tofe S., Vich F.; **Jaén:** Garijo J.M., Huete A., Lobón J.A., López J.A.; **La Rioja:** Brea A.J., Chinchetru M.J., Daroca R., Pérez S.; **Las Palmas:** Alberiche M.P., Flórez J., Lasso A., Marrero D., Rosquete J.E., Suárez S.; **León:** Chinea M., Fernández E., Marcos L.A., Suárez A.; **Lleida:** Baillo P., Torres G; **Lugo:** Fiallega J.R., Guerrero J., Vidal J.I.; **Madrid:** Artola S., Brito M.A., Calle A., Candel I., del Cañizo F.J., Coto A.L., Fernández A., Filgueira J.S., Garijo F.J., Gómez J.F., González C., González M.P., Guardiola E., Gutiérrez A., Guzón M.M., López-Hermosa P., Marrero J., Martínez J.L., Montoya T., Morón I., Muñoz M.V., Olivar J., Palacios N., Pinilla B., Rodríguez J.R., Rodríguez C., Saavedra M.P., Saiz F., Sanabria M.C., Serrano R., Utrilla V.; **Málaga:** Sedeno J., García J.A., García E., Maldonado J.A., Mancha I., Martínez J., Martos F.; **Melilla:** Quilez S.C.; **Murcia:** García J.A., García B., García-Galbis J.A., López J.F., Pascual M.H., Saucedo P.; **Ourense:** Fernández M.J., Suárez X.M., Vilarino C.R.; **Palencia:** Cuende J.M., Maldonado A., Pérez de Diego I.; **Pontevedra:** Fernández J.J., García A., García F.J., López G.; **Salamanca:** Miralles J.M.; **Tenerife:** Correa V.J., Dorta J.M., Maffiote N., Rodríguez M., Velasco J.L.; **Segovia:** Castro J.C., Moreno J.J., Roldán A.; **Sevilla:** Guerrero T., Contreras J., Griera J.L., Hernández C., Losada F., Lozano F., Montenegro L., Rico M.A., Sánchez J.; **Tarragona:** Corrales R., Marimón F., Pedrico D., Rabassa A.; **Teruel:** Gimeno J.A.; **Toledo:** Blanco B., Comas J.M., Puñal P.; **Valencia:** Artero A., Cervello G., Girbes-Borrás J.A., Godoy D., Martí J., Martínez C., Martínez I., Mateu C., Méndez J.M., Miquel L., Morata C., Muñoz M., Pastor J.A., Quinza V., Rodilla E., Sales J., Tamarit J.J., Tur M.D.; **Valladolid:** Asenjo I., del Valle M.A., García E., González E., Jimeno A., Zurro J.; **Zamora:** Gómez D., Núñez R.; **Zaragoza:** de Castro P., Gutiérrez G., Monreal M., Torrecilla J., Vidal C.
